# Making progress in early-career publishing: evolutions of the women’s publication mentorship programme

**DOI:** 10.1093/heapol/czad047

**Published:** 2023-11-16

**Authors:** Nanuka Jalaghonia, Aku Kwamie

**Affiliations:** Programme Manager, Health Systems Global, Canadian Association for Global Health, 75 Albert Street, Suite 1003, Ottawa, ON K1P 5E7, Canada; Technical Officer, Alliance for Health Policy and Systems Research, World Health Organization, 20 Avenue Appia, Geneva 27 1211, Switzerland

**Keywords:** Health systems, capacity strengthening, mentorship, women, science

## Abstract

Mentorship is vital for early-career researchers, especially women from low- and middle-income countries seeking to publish their work. This paper explores the evolution of the Women’s Publication Mentorship Programme, a collaborative initiative pioneered by the Alliance for Health Policy and Systems Research, further strengthened through the partnership of Health Systems Global, and Health Policy and Planning. Over a span of five years and encompassing three cohorts, the program supported 45 early-career researchers from 24 countries, resulting in insightful papers on equity-oriented health system topics. Beyond the direct outcomes of strengthening the writing skills of first-time women authors and facilitating paper publications, the Programme has also influenced Health System Global's strategic approach and conceptual framework for systemic capacity strengthening in health policy and systems research. It has also played a pivotal role in addressing the longstanding gender imbalance in global health authorship. Amid these achievements, our program consistently evolved, drawing from lessons of the past cohort. Challenges, such as the need for extended paper development timelines, addressing language barriers, and strengthening methodological rigor in initial manuscripts, were met with solutions. Insights and experiences from previous participants translated into tangible results, notably elevating the quality of journal supplement publications. This commentary explores key lessons from the second cohort's journey and its evolving nature. It also highlights persistent challenges and provides practical recommendations for organizations to enhance their mentorship programs, ultimately fostering the career growth of early-career researchers in health policy and systems research.

Key messages

- The Women’s Publication Mentorship Programme, launched in 2018, supports early-career mentorship for women seeking to publish their first manuscript and aims to empower early-career women in Low and Middle Income Countries (LMICs) by pairing them with experienced professionals in the field.
- The programme has supported 45 early-career researchers from 24 countries, addressed the persistent under-representation of women authors in global health, and informed Health Systems Global’s (HSG) approach and conceptual framework for systemic capacity strengthening.
- While developing manuscripts is a key output, the programme offers added value. A nascent foundation is being fostered to support lasting relationships that can accompany early-career researchers as they advance in their professional lives.
- There are three questions for the programme to consider as it continues to respond to the needs of Health Policy and Systems Research (HPSR) mentors and mentees:

1. How should such programmes think about developing mentoring skills in recent mentees to enable them to become mentors to future cohorts?
2. How should such programmes establish an alumnae network that can sustain a supportive mentoring environment for early-career researchers?
3. How should such programmes further encourage male allyship, through mentorship and other supporting roles?

As in all fields, the importance of mentorship for career progression in research, particularly in the early stages, cannot be overstated. This is especially true for women, particularly those from low- and middle-income countries (LMICs) who often face limited opportunities to have their work published. Formal mentorship programmes targeting women can help reduce gendered power dynamics and enable early-career researchers to gain knowledge from the experiences of others who have encountered similar obstacles (Brizuela *et al.*, 2023).

The publication mentorship programme, first launched by the Alliance for Health Policy and Systems Research (the Alliance) in 2018, is an initiative that supports early-career mentorship for women seeking to publish their first manuscript. Since 2020, the programme has developed as a three-way partnership between the Alliance, Health Systems Global (HSG) and Health Policy and Planning (HPP). The programme aims to empower early-career women in LMICs by pairing them with experienced professionals in the field.

Over the course of five years and three cohorts, the programme has supported 45 early-career researchers from 24 countries (Table 1), with a majority coming from Africa and Asia. In the two journal supplements published to date (with BMJ Global Health and HPP), various papers explore a wide range of equity-oriented topics affecting health system performance and outcomes. These topics, including health workforce support, intimate partner violence, health sector corruption, implementation factors in service delivery, universal health coverage, and more, are uniquely interpreted through the lenses of early-career women who live and work in these settings (Table 2). The publication mentorship programme has also informed HSG’s approach and conceptual framework for systemic capacity strengthening (Mirzoev *et al.*, 2022).

**Table 1.** Number and countries of mentees, 2018–22

Region	Country	Number of mentees
Africa	Benin	1
	Cameroun	1
	Ethiopia	1
	Ghana	1
	Liberia	1
	Kenya	5
	Malawi	2
	Nigeria	6
	South Africa	3
	Uganda	2
	Zambia	1
	Zimbabwe	2
Asia	Bangladesh	1
	India	4
	Indonesia	1
	Malaysia	1
	Pakistan	1
	The Philippines	3
	Sri Lanka	1
	Vietnam	1
Eastern Mediterranean	Yemen	1
Latin America and Caribbean	Brazil	2
	Colombia	1
	Peru	2
Total		**45**

The women’s publication mentorship programme addresses the persistent under-representation of women authors in global health. The literature shows that between 2002 and 2020, women represented only 39.3% of all global health authorship, 45.9% of these came from high-income countries, and only 28.2% from low-income countries (Yao *et al.*, 2022).

**Table 2.** Journal supplements, 2019–20

Journal supplement	Article title	Citation
*BMJ Global Health* * *2019;4(Suppl 10)	How local context influences access to neuropsychological rehabilitation after acquired brain injury in South Africa	(Joosub, 2019) 6 citations
	Sub-national perspectives on the implementation of a national community health worker programme in Gauteng Province, South Africa	(Munshi *et al.*, 2019) 11 citations
	Are formal self-care interventions for healthy people effective? A systematic review of the evidence	(Perera and Agboola, 2019) 15 citations
*Health Policy and Planning* 2020;**35**(Suppl 1)	Enhancing diversity in public health scholarship: the role of publication mentorship *(editorial)*	(Javadi and Hussain, 2020) 1 citations
	Supporting early-career mentorship for women in Health Policy and Systems Research: a vital input to building the field *(commentary)*	(Kwamie and Jalaghonia, 2020) 5 citations
	The magnitude of intimate partner violence during pregnancy in Eldoret, Kenya: exigency for policy action	(Luhumyo *et al.*, 2020) 3 citations
	Intimate partner violence is associated with poorer maternal mental health and breastfeeding practices in Bangladesh	(Tran *et al.*, 2020) 12 citations
	Factors associated with the utilization of inactivated polio vaccine among children aged 12 to 23 months in Kalungu District, Uganda	(Faith *et al.*, 2020) 2 citations
	Perspectives and practices of healthcare providers and caregivers on healthcare-associated infections in the neonatal intensive care units of two hospitals in Ghana	(Sunkwa-Mills *et al.*, 2020) 4 citations
	Predictors of nursing leadership in Uganda: a cross-sectional study	(Nanyonga *et al.*, 2020) 6 citations
	Effectiveness of a nutrition education and counselling training package on antenatal care: a cluster randomized controlled trial in Addis Ababa	(Omer *et al.*, 2020) 10 citations
	Do social accountability approaches work? A review of the literature from selected low- and middle-income countries in the WHO South-East Asia region	(Naher *et al.*, 2020) 5 citations
	How ready is the system to deliver primary healthcare? Results of a primary health facility assessment in Enugu State, Nigeria	(Ekenna *et al.*, 2020) 15 citations
	Health reform and Indigenous health policy in Brazil: contexts, actors and discourses	(de M Pontes and Santos, 2020) 17 citations

## Learning for improvement: adapting the women’s publication mentorship programme

In 2020, the authors of the present article wrote a commentary highlighting the critical need for mentorship opportunities for early-career women researchers. This was based on insights gained from the second cohort of the mentorship programme (Kwamie and Jalaghonia, 2020). The challenges raised in that paper included reflections on the programme’s short timelines, language barriers and (in some cases) methodologically weak initial manuscripts.

Eliciting past participants’ feedback, and drawing on these learnings, we made improvements to the third cohort of the programme. These included: extending the paper development phase to allow mentees to fully absorb the skills being learned; introducing a joint orientation process, which entails conducting an onboarding webinar and providing supporting materials that include information about roles and responsibilities, expectations of each party involved in the mentorship program, and the timeline; increasing regular communications with the mentor–mentee pairs through program organizers; scheduling more frequent check-ins to monitor progress, including an interim paper review by the co-editors of this supplement and added support from the journal (including submission guidelines and editorial assistance).

In this commentary, we delve into the insights we gained from the second cohort of the programme, highlighting the key lessons learned and how the programme has evolved over time. We also identify the remaining challenges that require attention. Based on our findings, we derive a set of practical recommendations that organizations can implement to enhance their mentorship programmes and support career growth.

## Reflecting on previous cohorts: what has improved, what is still a challenge?

We conducted a short survey of mentors and mentees in the current cohort (*n* = 26, 13 mentees, 13 mentors), with a response rate of 81% (21 out of 26 people), to gain insights into their perspectives. While the sample size is modest, the high response rate suggests that the results are representative of most participants.

The survey underscored the importance of the professional development opportunities that the programme provides for both mentees and mentors. For mentees, this went beyond simply enhancing their scientific writing skills, towards strengthening ‘soft skills’ such as critical thinking, communication, time management, and gaining appreciation and confidence to participate in the field of HPSR as a peer. For mentors, they gained valuable insights into developing stronger mentoring skills. These changes had a positive impact on the development of papers leading to higher quality publications in the journal supplement.

Although face-to-face meetings for mentor–mentee pairs at HSG Global Symposia on Health Systems Research (HSR) has been part of the programme design since 2019, COVID-19 travel restrictions meant that this did not take place at HSR 2020 (HSR, 2020). However, it did happen at HSR 2022 (HSR, 2022), and the energy released during these face-to-face meetings emphasized just how important such opportunities are for mentorship. The survey results showed that face-to-face meetings led to better communication and collaboration on completing the manuscripts. Furthermore, the opportunity to work across mentor–mentee pairs increased learning for both mentors and mentees as fresh conceptual or methodological perspectives were brought to bear. This created a stronger sense of community within the programme. For subsequent cohorts, mentors and mentees suggested conducting longer face-to-face meetings at the beginning and in the middle of the programme to foster relationships and enhance learning further.

Despite program improvements, including an extension of the paper development period by three months, some challenges persisted. As first-time authors without prior publication experience, mentees found it difficult to develop their scientific papers within the given time frame. Short timelines remained an issue for mentees in the program.

As first-time authors without prior publication experience, mentees found it difficult to develop their scientific papers within the given time frame. The low publication rate of our programme, with only 13 papers published among 45 manuscripts over three cohorts, highlights the systemic and societal problems that many early-career women face in research, particularly in LMICs. These challenges include limited time for manuscript development, inadequate access to research databases, and organizational cultures that discourage mentorship; therefore, more programmes that provide essential resources, training and mentorship are necessary to support the success of early-career women in research.

According to feedback from our mentees in a recent survey, more programme time dedicated to manuscript development and training in paper writing is essential for producing higher-quality manuscripts. We have also observed that intensifying peer review and feedback, as well as fostering more frequent communication and setting achievable publication goals between mentors and mentees, can be effective strategies to address the persistent difficulties faced by mentees in producing manuscripts with a greater likelihood of publication. The literature also supports these observations and emphasizes the importance of mentor experience, availability, alignment of research interests with mentee topics, and a strong commitment to supporting mentees to achieve their objectives and ensure their success (Diggs-Andrews *et al.*, 2021).

Another challenge is the perception of initial manuscripts that are sometimes methodologically weak. Suggestions from the cohort for the future include: a more rigorous initial abstract screening procedure, although this in some ways contradicts the capacity strengthening objectives of such a programme (i.e. manuscripts are submitted for mentoring precisely because the authors need to be coached on strengthening their submissions); or possibly initiating a ‘pre-mentorship preparatory’ phase, which would support potential applicants with some training and guidance prior to submitting their abstracts to the programme for selection.

## What is next for the programme?

As the publication mentorship programme continues to mature, new reflections are emerging from the community that is being formed by the previous cohorts. While developing manuscripts is a key output, the programme offers added value. We observe a nascent foundation being fostered to support lasting relationships that can accompany early-career researchers as they advance in their professional lives. Anecdotally, earlier mentor–mentee pairs have reported that they have continued to collaborate on other manuscripts as a result of this programme, and that mentors have recommended their mentees for other leadership positions. To create further opportunities for cohort engagement, a LinkedIn group was recently formed.

There are three other considerations for the programme as it continues to respond to the needs of HPSR mentors and mentees. First, how should such programs think about developing mentoring skills in recent mentees to enable them to become mentors to future cohorts? Current mentees have expressed a strong interest in participating in the program again, but this time as mentors to ‘pay it forward’. This emphasizes the importance of providing evolving capacity-strengthening opportunities to help them take on these roles with confidence and competence, as recommended by the World Health Organization collaboration in *Health research mentorship in low and middle-income countries (HERMES)* (World Health Organization (WHO), 2022a).

To achieve this goal, it is important to build the programme’s internal capacity to train mentees in the technical skills of mentoring and establish a follow-up phase to the publication mentorship programme that prioritizes this aspect. Although this may take time and require increased resources, investing in the development of a follow-up phase has the potential to create a community of experienced mentors who can guide future generations of mentees in HPSR, thereby contributing to the growth and development of the field in a sustainable manner. Strengthening individual capacities through mentoring also has the potential to contribute at an organizational level by ensuring that mentees and mentors are equipped to continue mentoring in their home institutions.

Second, how should such programmes establish an alumnae network that can sustain a supportive mentoring environment for early-career researchers? Given that less than a third of pairs have published papers from the scheme so far, establishing such a network could facilitate ongoing professional development. HSG is well positioned to support and sustain this mentoring network given its commitment to supporting HPSR communities, and through its 6 regional networks and 10 thematic working groups. Many participants in the current cohort expressed a strong interest in joining a supportive alumnae network.

Last, how should such programmes further encourage male allyship, through mentorship and other supporting roles? While recognizing the invaluable contributions of female mentorship, it is equally important not to propagate further imbalances in women’s ‘unpaid labour’ by developing a programme that is based on women’s volunteer work. Over the three cohorts, only a minority of mentors have been men. This observation is consistent with research showing that women are often expected to assume more informal and unpaid responsibilities, including volunteering, while men do not always face the same expectations (Ferrant *et al.*, 2014; World Health Organization (WHO), 2019; 2020b).

The low rate of male mentors in our programme may also be due to a lack of intentional efforts to recruit them, leading to an assumption that the programme is primarily for women. To create a more inclusive environment for all participants, future programmes should explicitly invite male mentors to participate and make more intentional efforts to recruit them, while also considering additional incentives to reward and incentivize both female and male mentors.

However, expanding mentorship programmes poses new obstacles, including the need for more resources, maintaining consistent quality and standards, and coordinating communication with a larger group of participants (Woltering *et al.*, 2019). It is important to note that such a capacity strengthening initiative needs dedicated and long-term funding to ensure its sustainability and effectiveness. Short-term, ad hoc funding may compromise the quality and impact of the programme. Therefore, a clear vision for the programme’s long-term goals and sustained investment are necessary to achieve the desired outcomes.

The publication mentorship programme has shown that a small-scale initiative to support early-career women in publishing their research can yield multiple benefits, beyond individual mentees and mentors, by facilitating a community of early-career researchers, and contributing to the development of the field of HPSR. Further learning from this programme can serve as a useful model to other capacity strengthening programmes in the future.

## Reflexivity statement

Our authorship team reflects diverse perspectives, with two mid-level female authors with local and global experiences in research and management. One author (N.J.) brings practical experience in public health and health policy in Georgia, while the other (A.K.) is a health policy and systems researcher with expertise in applying complexity theory to health systems governance, management and leadership in Africa and globally. Both authors have experience in conducting qualitative research in low- and middle-income settings.
